# Endoscopic therapy for patients with pancreaticobiliary maljunction: a follow-up study

**DOI:** 10.18632/oncotarget.16228

**Published:** 2017-03-15

**Authors:** Zheng Jin, Li-Ke Bie, Yan-Ping Tang, Liang Ge, Si-Si Shen, Bin Xu, Tao Li, Biao Gong

**Affiliations:** ^1^ Department of Gastroenterology, Digestive Endoscopy Center, Ruijin Hospital, Shanghai Jiaotong University School of Medicine, Shanghai, China; ^2^ First People’s Hospital of Hangzhou, Hangzhou, Zhejiang, China; ^3^ Department of Gastroenterology, Shihezi People’s Hospital, Shihezi, China; ^4^ The Second Affiliated Hospital, Zhejiang University School of Medicine, Hangzhou, Zhejiang, China

**Keywords:** pancreas divisum, endoscopic retrograde cholangiopancreatography, endoscopic pancreatic sphincterotomy, endoscopic nasopancreatic drainage, endoscopic retrograde pancreatic drainage

## Abstract

**Background:**

Data on the experience of endoscopic retrograde cholangiopancreatography (ERCP) in the management of pancreaticobiliary maljunction (PBM) is limited.

**Methods:**

A retrospective review of patients with PBM who underwent therapeutic ERCP at our endoscopy center between January 2008 and January 2016 was performed. Demographic, clinical, radiological and endoscopic data was documented. Patients who underwent sphincterotomy were divided into dilated group and undilated group based on their common channel diameter.

**Results:**

Sixty-three PBM patients underwent 74 ERCP procedures. The technical success rate was 97.3%. ERCP therapy significantly decreased the levels of elevated liver enzymes and bilirubin. After an average of 27 months follow-up, 7 patients (11.1%) were lost. The overall effective rate of ERCP therapy was 60.7% (34/56). Decline in severity and frequency of abdominal pain was significant. Procedure-related complications were observed in 5 (6.8%) cases. Between the dilated group and undilated group, no significant difference was observed in effective rate, adverse events and follow-up results.

**Conclusions:**

ERCP can serve as a transitional step to stabilize PBM patients before definitive surgery. PBM patients with undilated common channel could benefit from sphincterotomy as well as those with dilated common channel.

## INTRODUCTION

Pancreaticobiliary maljunction (PBM) is a congenital anomaly in which the pancreatic duct and bile duct join together and form a long common channel outside the duodenal wall [1]. Without the influence of sphincter of Oddi, pancreatic juice and bile regurgitate and mix, producing various pathological conditions such as congenital biliary dilatation, pancreatitis, protein plugs and biliary carcinoma. It's consensus to perform a prophylactic surgery for PBM patients as soon as possible when the diagnosis is made [1]. However, patients of PBM are always accompanied by obstructive jaundice or acute pancreatitis, surgery in these patients is thought to increase post-operative risk. Preoperative endoscopic retrograde cholangiopancreatography (ERCP) may improve drainage, resolve complications and allow for a subsequent safe operation.

Nonetheless, Data on the experience of ERCP in the management of PBM are limited to small series [2–10]. Two studies speculated that endoscopic sphincterotomy (EST) was less effective in those patients with undilated common channel, but their sample size was quite small [2, 7]. Therefore, we conducted this retrospective study to identity (1) the efficacy and safety of ERCP in the therapy of PBM and (2) the difference of prognosis between patients with dilated common channel and undilated common channel after EST.

## MATERIALS AND METHODS

### Patient selection

From January 2008 to January 2016, consecutive patients with PBM who had undergone endoscopic therapy in the digestive endoscopy center of our hospital were included.

Inclusion criteria contain: 1) PBM confirmed by ERCP, 2) ERCP treatment performed. Exclusion criteria include: 1) patients with primary sclerosing cholangitis, malignant diseases or prior liver transplantation; 2) therapeutic ERCP was not performed.

In patients who had repeated ERCP therapy, each ERCP was regarded as an independent case (i.e. number of cases > number of patients). Demographic, clinical, radiological and endoscopic data of the included patients was obtained from medical records. Data on pre- and post-ERCP laboratory parameters including serum alanine aminotransferase (ALT, normal range: 10-64 IU/L), aspartate aminotransferase (AST, normal range: 8-40 IU/L), alkaline phosphatase (ALP, normal range: 38-126 IU/L), γ-glutamyl transpeptidase (γ-GT, normal range: 7-64 IU/L), total bilirubin (TBIL, normal range: < 24 μmol/L), direct bilirubin (DBIL, normal range: < 6.8 μmol/L) and amylase (normal range: 28-100 IU/L) concentrations was collected within seven days before and after the day of ERCP, respectively. Only patients with abnormal laboratory data before ERCP were analyzed.

### ERCP procedures

Informed consent was obtained after the risks and benefits of the ERCP were explained to the patient and key family members. A subsequent radical surgery was recommended to all patients considering the risk of malignancy. All ERCPs were performed under general anesthesia with endotracheal intubation, in the prone position, with a duodenoscope by two experienced endoscopists (BG and LKB). Duodenoscope JF240 (tip outer diameter, 12.6 mm; channel diameter, 3.2 mm; Olympus, Japan) was used for infants and children, JF-260V (tip outer diameter, 12.6 mm; channel diameter, 3.7 mm; Olympus, Japan) or TJF-260V (tip outer diameter, 13.5 mm; channel diameter, 4.2 mm; Olympus, Japan) were used for adults.

We first attempted common channel cannulation using a doublelumen sphincterotome (5.5F; Endo-Flex, Germany), and obtained an optimal image of pancreaticobiliary junction. Once a definitive diagnosis of PBM was established, sphincterotome preloaded with a guidewire (0.035mm; Innovex, China) was used for selective cannulation. Pre-cut method was applied in cannulation failed cases. After successful cannulation of common bile duct (CBD), we aspirated 10 ml of bile sample for measurement of biliary amylase (Bile was not obtained in all the patients because of endoscopist’ s slip). EST was performed to help the bile and pancreatic juice flow freely into the duodenum. Epinephrine-containing icy saline (1:10,000) was injected into the submucous coat of the papilla to prevent post-EST hemorrhage. We also used endoscopic hemoclip placement (EHP) to treat patients with high risk of post-EST bleeding. Strictures were dilated by biliary dilation catheters (6 - 8.5F, Cook, USA) and/or balloons (12-18mm, Innovex, China). Stones were extracted with baskets (Micro-tech, China) and/or balloons (12-18mm, Innovex, China). Endoscopic papillary balloon dilation (EPBD) were applied if a large CBD stone was present. At last, endoscopic nasobiliary/nasopancreatic drainage (ENBD/ENPD) or endoscopic retrograde biliary/pancreatic drainage (ERBD/ERPD) was performed to prevent complications when it was necessary. After ERCP, the American Society for Gastrointestinal Endoscopy (ASGE) grading system was used to grade the complexity of ERCP procedures [11].

### Follow-up

The following parameters were assessed by phone calls and by searching the medical records for the period from the initial ERCP to the radical surgery (if available) or to the last follow-up: general condition (5-point Likert scale: 1=excellent; 2=better; 3=same; 4=worse; and 5=much worse), severity of abdominal pain (0-10 visual analogue scale: 0 = no pain, 10 = extreme pain), frequency of abdominal pain/pancreatitis (0 = never, l = yearly, 2 = monthly, 3 = weekly, 4 = daily, 5 = continuously), number of readmission, and number of added ERCP. A comparison of the patient condition before and after the index ERCP therapy were analyzed to evaluate the follow-up outcome.

### Definition

**Figure 1:**
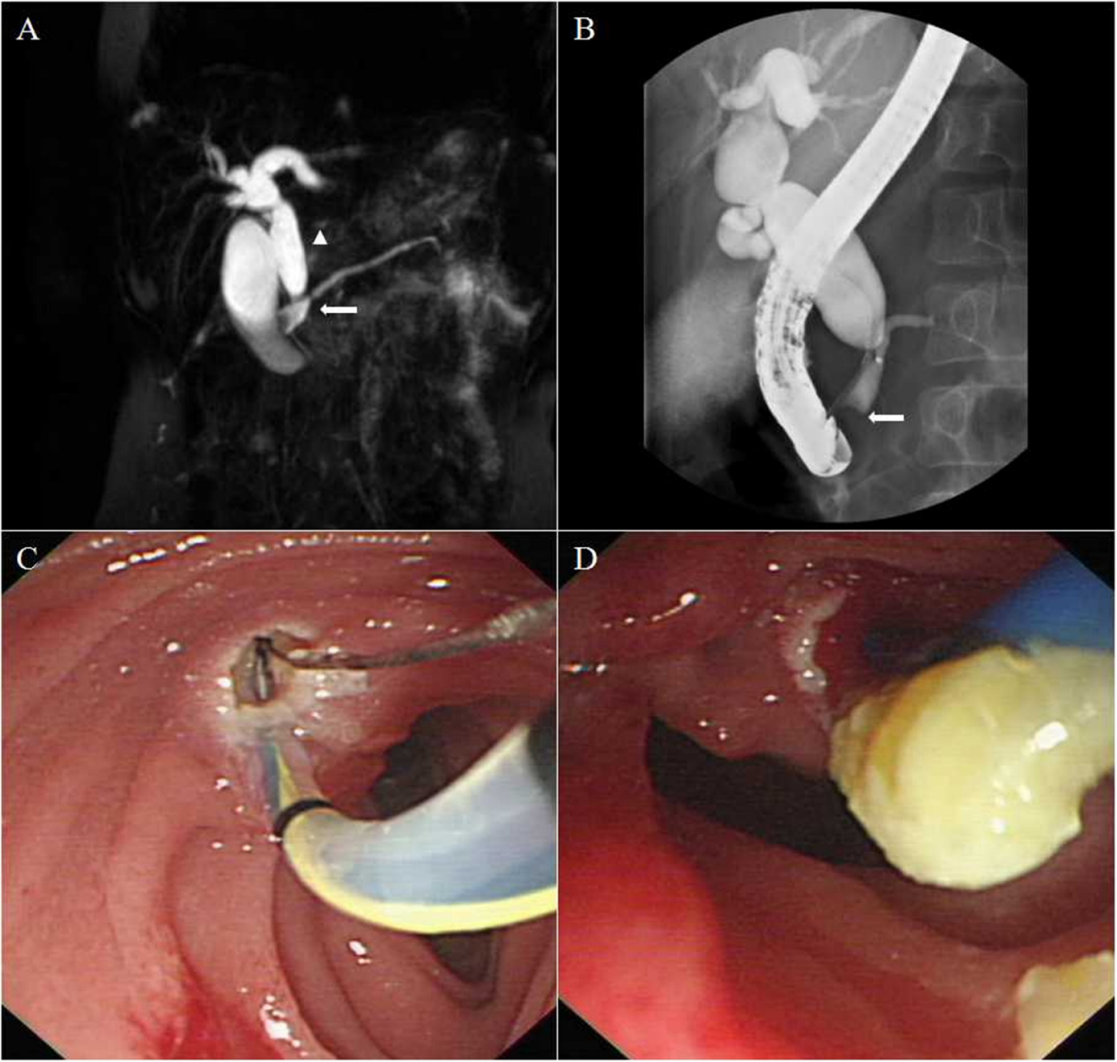
Representative images of a patient with a Komi type a PBM **A**. MRCP showing a Todani type IV-A dilated common bile duct (arrow head) joined the pancreatic duct at a right angle (arrow). **B**. ERCP confirming a PBM forming a common channel for a length of up to 2cm. Protein plugs were detected as filling defects located in the dilated common channel (arrow). **C**. Endoscopic view of sphincterotomy through the major papilla. **D**. Protein plugs extraction.

The diagnostic criteria for PBM were (1) An abnormally long common channel and/or an abnormal union between the pancreatic and bile ducts must be evident on direct cholangiography. (2) In cases with a relatively short common channel, it is necessary to confirm that the effect of the papillary sphincter does not extend to the junction by direct cholangiography. (3) Abnormally high levels of pancreatic enzymes in the bile duct and/or the gallbladder serve as an auxiliary diagnosis [12]. PBM was classified into three types according to Komi’ s classification [13]. Congenital biliary dilatation was defined as congenital dilatation of the extrahepatic or intrahepatic bile ducts or both (adults > 10 mm, pediatric patients > 6 mm), and had been classified into five types by Todani [14]. A dilated common channel was defined by the common channel > 5mm in diameter [1]. Protein plugs, which resulted from the retention of pancreatic juice mixed with bile, were fragile in texture and translucent on cholangiopancreatography [15] (Figure 1). ERCP technical success was assessed based on the intent of the ERCP procedure, which is usually known before the procedure. ERCP therapy was considered to be effective if patient reported improvement of general condition (better or excellent) after his index ERCP, without having required readmission or further endoscopic intervention. Adverse events (AEs) were assessed on the basis of the consensus criteria [16].

### Statistical analysis

SPSS Statistics 18.0 software was used. Categorical variables were expressed as frequencies and percentages and continuous variables were expressed as means with standard deviation (SD) or range. The Wilcoxon signed-rank test (for paired samples) and Mann-Whitney test (for unpaired samples) were used for the comparison of continuous data. The categorical variables were tested using χ^2^ test with Yates correction or with Fisher's Exact Test. Statistical significance was defined as *p* < 0.05 (two-tailed). The study protocol was approved by the Institutional Review Board in our hospital.

## RESULTS

### Patient characteristics

A total of 63 PBM patients underwent 74 ERCP treatments (*n* = 74 cases). Forty-five (71.4%) patients were female. The mean age was 24 (range 1 - 82) years, 38 patients (60.3%) were under 18 years of age. Among them, 37 patients were under 12 (age was ≤ 1 year in 2 patients, 1 - 12 years in 35 patients), 1 patient was adolescent (aged 13 - 17 years). The main symptoms of the cases (one case may involve one or more symptoms) included abdominal pain (*n* = 69, 93.2%), vomiting (*n* = 35, 47.3%), jaundice (*n* = 9, 12.2%), and fever (*n* = 9, 12.2%). The mean duration of symptom before treatment was 2.5months (range 4 days-20 years). Indications for ERCP (one case may involve one or more indications) were pancreatitis (*n* = 28, 37.8%, 20 children and 8 adults), pancreaticobiliary calculi (*n* = 48, 64.9%, 34 children and 14 adults), biliary obstruction (*n* = 19, 25.7%, 6 children and 13 adults), and stent migration (*n* = 1, 1.3%, 1 adult).

### PBM features

**Figure 2:**
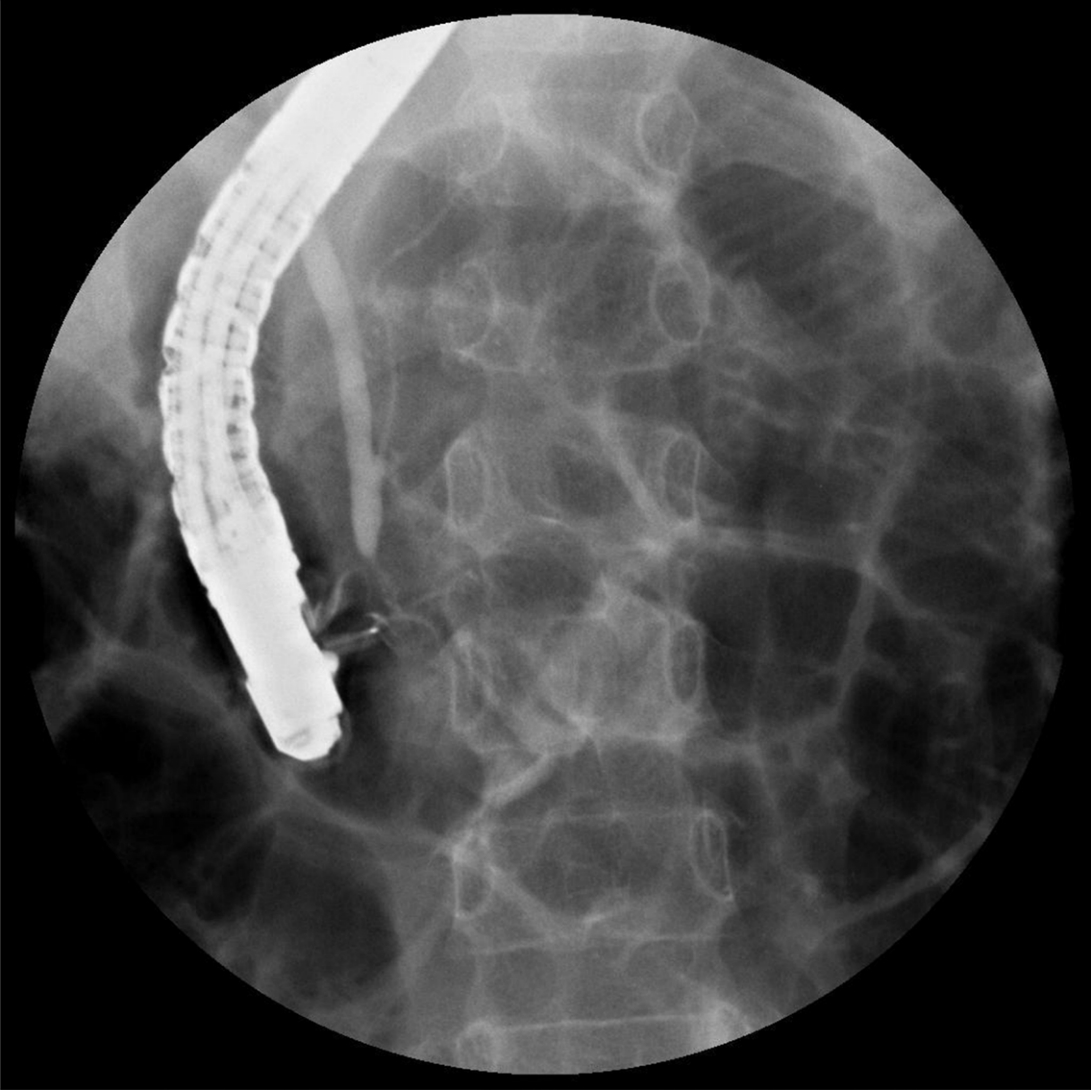
Representative fluoroscopic image of a patient with a Komi type b PBM The pancreatic duct joined the undilated common bile duct at an acute angle forming a common channel for a length of 1.5 cm.

**Figure 3:**
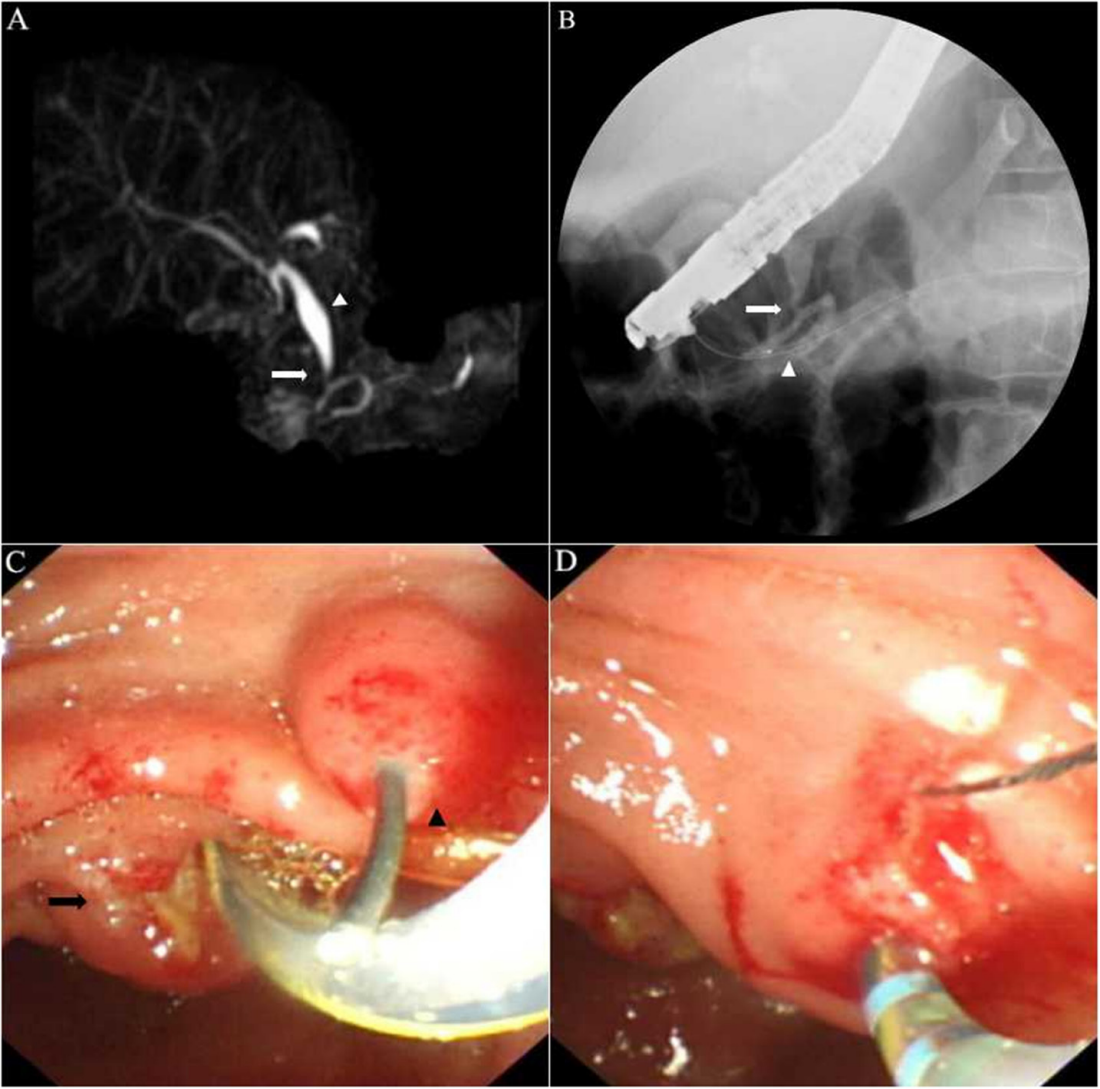
Representative images of a patient with a Komi type c PBM **A**. MRCP showing a common bile duct (CBD) with Todani type Ic dilation (arrow head) seemed to join dorsal pancreatic duct at 1.7 cm from the minor papillary orifice. The bizarre course of the pancreatic ducts suggested incomplete pancreas divisum. **B**. Fluoroscopic view of ERCP: After cannulation of the minor papilla, injection of contrast medium into the dorsal pancreatic duct showed that the CBD joined a side branch of the dorsal pancreatic duct instead (arrow), not the dorsal pancreatic duct itself (arrow head). **C**. Endoscopic view showing a guidewire went into the major papilla (arrow) and went out from the minor papilla (arrow head), which demonstrated the existence of incomplete pancreas divisum. **D**. Sphincterotomy through the major papilla and the minor papilla, respectively.

ERCP showed Komi type a PBM in 36 patients (57.1%, Figure 1), type b in 24 patients (38.1%, Figure 2) and type c in 3 patients (4.8%, Figure 3). Thirty-four patients (54.0%) had congenital biliary dilatation. Per Todani’ s classification, 12 (19.0%) had type Ia, 11 (17.5%) had type Ic, and 11 (17.5%) had type IV-A. The mean length of the common channels was 16.1 mm, ranging from 6.1 to 27.9 mm (< 15 mm in 30 patients), 23 of them (36.5%) were dilated. For pediatric patients, the mean length of the common channels was 14.9 mm, ranging from 6.1 to 27.9 mm (< 15 mm in 18 patients), 15 of them (36.5%) were dilated. Twenty-one cases (28.4%, 17 children and 4 adults) were found to have protein plugs in their common channels (*n* = 13), or in the pancreaticobiliary ducts adjacent to the common channel (*n* = 8). Eighteen cases (24.3%, 10 children and 8 adults) had extrahepatic bile duct stones, 9 (12.2%, 7 children and 2 adults) had pancreatic stones. Three patients were complicated by pancreas divisum (2 incomplete and 1 complete), 1 patient had low confluence of cystic duct and CBD, 6 patients suffered with chronic pancreatitis. The mean level of biliary amylase of 23 patients whose bile was obtained was 55,716 (range 1222 to 353269) IU/L. Only two patients’ biliary amylase concentration was under 10000 IU/L. They were both adults, one had severe jaundice, the other had prior EST treatment. Table 1 summarized the baseline demographic and clinical characteristics of study patients.

**Table 1:** Baseline characteristics of included patients

Age, mean (range), years	24 (1-82)
Females, n (%)	45 (71.4)
Symptoms*, n (%)	
Abdominal pain, n (%)	69 (93.2)
Vomiting	35 (47.3)
Jaundice	9 (12.2)
Fever	9 (12.2)
Indications*, n (%)	
Pancreatitis	28 (37.8)
Pancreaticobiliary calculi	48 (64.9)
Protein plugs	21 (28.4)
Extrahepatic bile duct stones	18 (24.3)
Pancreatic stones	9 (12.2)
Biliary obstruction	19 (25.7)
Stent migration	1 (1.3)
Type of PBM, n (%)	
Komi type a	36 (57.1)
Komi type b	24 (38.1)
Komi type c	3 (4.8)
Congenital biliary dilatation, n (%)	
None	29 (46.0)
Todani type Ia	12 (19.0)
Todani type Ic	11 (17.5)
Todani type IV-A	11 (17.5)
Length of common channel, mean (range), mm	16 (6.1-27.9)
Common channel dilation, n (%)	23 (36.5)
Biliary amylase, mean (range), IU/L	55,716 (1,222-353,269)
Accompanied diseases, n (%)	
Pancreas divisum	3 (4.8)
Low confluence of cystic duct and CBD	1 (1.6)
Chronic pancreatitis	6 (9.5)

* One case may involve one or more indications.

### Therapeutic interventions

Therapeutic ERCPs were performed in all 74 cases (range 1 to 3 times per patient). The technical success rate was 97.3% (72/74). Pancreatic stones could not be extracted in two cases because of complicated pancreaticobiliary ductal union. Therapeutic procedures included pre-cut in 1 case (1.4%), EST in 57 cases (77.0%, EST for both the major and minor papilla in one case), EHP in 4 cases (5.4%), EPBD in 11 cases (14.9%), stricture dilation in 5 cases (6.8%), stone extraction in 46 cases (62.2%), ERBD in 15 cases (20.3%, two stents inserted simultaneously in one case), ERPD in 4 cases (5.4%, ERPD through the minor papilla in one case), ENBD in 48 cases (64.9%) and ENPD in 3 cases (4.1%). The procedure difficulty was determined as 1^st^ level in 17 cases (23.0%), 2^nd^ level in 29 (39.2%), 3^rd^ level in 23 (31.1%), and 4^th^ level in 5 (6.7%).

### Clinical outcome

The levels of serum ALT (186.9 ± 106.6 vs. 64.8 ± 39.4, *p* < 0.001), AST (148.8 ± 91.1 vs. 46.3 ± 28.5, *p* < 0.001), ALP (381.6 ± 122.9 vs. 261.5 ± 84.7, *p* < 0.001), γ-GT (340.3 ± 168.8 vs. 215.2 ± 91.1, *p* < 0.001), TBIL (52.7 ± 41.6 vs. 24.8 ± 19.2, *p* < 0.001) and DBIL (24.1 ± 14.2 vs. 10.1 ± 9.3, *p* = 0.003) were remarkably decreased after ERCP therapy (Table 2). A trend towards increase in the level of serum amylase was observed, but the change was not statistically significant (244.7 ± 204.0 vs. 307.6 ± 356.0, *p* = 0.6). The mean (± SD) duration of hospitalization after ERCP was 6.3 (± 2.8) days.

**Table 2:** Parameters comparison between pre-ERCP and post-ERCP

**Details**	**Pre-ERCP**	**Post-ERCP**	***p* Value**
Serum biochemistry			
ALT, IU/L	186.9 ± 106.6	64.8 ± 39.4	<0.001
AST, IU/L	148.8 ± 91.1	46.3 ± 28.5	<0.001
ALP, IU/L	381.6 ± 122.9	261.5 ± 84.7	<0.001
γ-GT, IU/L	340.3 ± 168.8	215.2 ± 91.1	<0.001
TBIL,μmol/L	52.7 ± 41.6	24.8 ± 19.2	<0.001
DBIL,μmol/L	24.1 ± 14.2	10.1 ± 9.3	0.003
Amylase, IU/L	244.7 ± 204.0	307.6 ± 356.0	0.6
Severity of pain	7.0 ± 3.2	0.9 ± 1.9	<0.001
Frequency of pain	1.8 ± 1.3	0.6 ± 1.1	<0.001
Frequency of pancreatitis	0.5 ± 0.8	0.3 ± 0.5	0.103

Data expressed as mean ± standard deviation (SD).

ALT, alanine aminotransferase; AST, aspartate aminotransferase; ALP, alkaline phosphatase; γ-GT, γ-glutamyl transpeptidase; TBIL, total bilirubin; DBIL, direct bilirubin; AMY, amylase.

During an average of 27 months (range 14 days - 82 months) follow-up, 7 patients (11.1%) were lost. In the remaining 56 patients, 12 (21.4%) had radical surgery after an average of 13 months (range 14 days - 65 months). Two patients (3.6%) developed gallbladder carcinoma at follow-up. They were both female in their sixties, without congenital biliary dilatation. One had type a PBM, whose cancer was discovered 3 years after ERCP. The other had type b PBM, whose cancer was discovered during the prophylactic cholecystectomy 3 months after ERCP.

The total effective rate of ERCP therapy in PBM was 60.7% (34/56). Among whom, 7 patients underwent radical operation with soundly preoperative condition, 27 patients without surgery were still in good clinical condition. Endoscopic intervention resulted in significant decline in severity (7.0 ± 3.2 vs. 0.9 ± 1.9, *p* < 0.001) and frequency (1.8 ± 1.3 vs. 0.6 ± 1.1, *p* < 0.001) of abdominal pain, but no significant change in frequency of pancreatitis (0.5 ± 0.8 vs. 0.3 ± 0.5, *p* = 0.103) was observed. Fifty patients (89.3%) reported their general condition after index ERCP were excellent or better, 5 (8.9%) were same, and 1 (1.8%) was worse. Eighteen patients (32.1%) required readmission (range 1 to 3 times per patient) and 10 patients (17.9%) underwent added ERCP (range 1 to 2 times per patient). The follow-up results were detailed in Table 3

**Table 3:** Details of follow-up results

**Details**	***n* = 56 patients**
Follow-up loss, n (%)	7 (11.1)
Follow-up duration, mean ± SD, months	26.7 ± 22.8
The duration of hospitalization after ERCP, mean ± SD, days	6.3 ± 2.8
General condition, mean ± SD	1.5 ± 0.7
Effective rate, n (%)	34 (60.7)
Patients who underwent radical surgery, n (%)	12 (21.4)
The duration from index ERCP to radical surgery, mean ± SD, months	13.0 ± 19.4
Patients who required readmission, n (%)	18 (32.1)
Patients who underwent further ERCP therapy, n (%)	10 (17.9)

SD, standard deviation.

### Adverse events

Post-ERCP AEs were observed in 5 (6.8%) cases, 3 (4.1%) were diagnosed with moderate post-ERCP pancreatitis (PEP) and 2 (2.7%) were mild hemorrhage. All AEs were resolved completely by conservative treatment. No patient had to be taken to the intensive care unit, and hospital mortality was zero.

### Further analysis

In our study, 56 patients underwent 57 EST procedures and 6 were excluded from the analysis due to follow-up loss. We therefore categorized 50 patients into two groups according to whether their common channels were dilated or not: the dilated group (*n* = 20; mean [range] age, 25 [1 - 78] years; 15 women [75.0%]) and the undilated group (*n* = 30; mean [range] age, 23 [1 - 82] years; 22 women [73.3%]). There was no statistical difference between two groups for baseline data. After ERCP therapy, no difference of severity (1.45 ± 2.28 vs. 0.73 ± 1.66, *p* = 0.353) and frequency (0.5 ± 0.76 vs. 0.73 ± 1.31, *p* = 0.748) of abdominal pain was identified between two groups. Frequency of pancreatitis (0.3 ± 0.57 vs. 0.27 ± 0.52, *p* = 0.873) did not differ between two groups either. No difference was seen for effective rate (14 [70.0%] vs. 21 [70.0%], *p* > 0.05), general condition (1.35 ± 0.59 vs. 1.53 ± 0.73, *p* = 0.401), number of readmission (6 [30.0%] vs. 9 [30.0%]; *p* > 0.05) and number of added ERCP (5 [25.0%] vs. 4 [13.3%]; *p* = 0.499). Regarding adverse events, no statistical difference was observed between two groups (0 [0%] vs. 2 [4.0%]; *p* = 0.510). The results of further analysis were detailed in Table 4.

**Table 4:** Baseline characteristics and follow-up results between two groups

**Details**	**Dilated** (***n*= 20)**	**Undilated** (***n*= 30)**	***p* value**
Age, mean (range), years	25 (1-78)	23 (1-82)	0.812
Females, n (%)	15 (75.0)	22 (73.3)	0.895
Type of PBM, n (%)			0.544*
Komi type a	13 (65.0)	15 (50.0)	
Komi type b	7 (35.0)	14 (46.7)	
Komi type c	0 (0)	1 (3.3)	
Common channel length, mean ± SD, mm	16.3 ± 5.4	14.9 ± 4.8	0.342
Severity of pain before index ERCP, mean ± SD	7.25 ± 2.67	6.73 ± 3.37	0.738
Frequency of pain before index ERCP, mean ± SD	2.15 ± 1.39	1.53 ± 1.14	0.127
Frequency of pancreatitis before index ERCP, mean ± SD	0.45 ± 0.89	0.5 ± 0.82	0.735
Follow-up results			
Follow-up loss, n (%)	1 (5.0)	5 (16.7)	0.424
Follow-up duration, mean ± SD, months	37.15 ± 20.46	29.37 ± 22.07	0.191
Duration of hospitalization after ERCP, mean ± SD, days	5.9 ± 1.9	6.1 ± 3.3	0.984
General condition, mean ± SD	1.35 ± 0.59	1.53 ± 0.73	0.401
Effective rate, n (%)	14 (70.0)	21 (70.0)	> 0.05
Patients who underwent readmission, n (%)	6 (30.0)	9 (30.0)	> 0.05
Patients who underwent further ERCP therapy, n (%)	5 (25.0)	4 (13.3)	0.499
Severity of pain after index ERCP, mean ± SD	1.45 ± 2.28	0.73 ± 1.66	0.353
Frequency of pain after index ERCP, mean ± SD	0.5 ± 0.76	0.73 ± 1.31	0.748
Frequency of pancreatitis after index ERCP, mean ± SD	0.3 ± 0.57	0.27 ± 0.52	0.873
Adverse events, n (%)	0 (0)	2 (4.0)	0.510
Hemorrhage	0 (0)	2 (4.0)	0.510

SD, standard deviation; *tested using χ^2^ test of multiple constituent ratios.

## DISCUSSION

In our study, PBM showed a female predominance (71.4%), and a higher percentage (60.3%) in pediatric patients. The main pre-ERCP complications were pancreaticobiliary calculi, biliary obstruction and pancreatitis. Regarding the Komi’ s classification of PBM, type a was more frequent than other two types. Congenital biliary dilatation was present in 54% of the PBM patients and Todani type Ia, Ic and IV-A were detected most commonly. Compared with the survey carried out by the JSPBM in Japan [17], the characteristics of PBM in our study were similar in general, with few difference in proportion. The length of the common channel is not included in the diagnostic criteria for PBM [12]. Many would consider a common channel longer than 15 mm to be abnormal [9, 18]. In our series, the mean length of the common channels was 16.1 mm, ranging from 6.1 to 27.9 mm. Thirty patients (47.6%) showed a common channel length < 15 mm. This was a relatively high percentage probably because most PBM patients with obviously long common channels can be diagnosed by MRCP. If they didn't need endoscopic therapy, they would be excluded from our study. It was reported that the biliary amylase levels in PBM were often at least 10,000 IU/L [17]. In our study, the mean level of biliary amylase was 55,716 (range 1222 to 353269) IU/L. Only two adult patients’ biliary amylase concentration was under 10000 IU/L (1222 and 5746 IU/L), one had severe jaundice, the other had prior EST treatment. Two possibilities may explain this phenomenon. First, the pancreatic juice reflux reduced due to the severe biliary obstruction [19]. Second, prior EST established free drainage and had a positive effect. Further investigation is still necessary. Protein plugs compacted in the common channel are thought to represent the cause of the symptoms and even acute pancreatitis in PBM [15]. The JSPBM guideline mentioned that protein plugs were detected in at least 30 % of pediatric PBM patients [1]. In our series, 28.4% (21/74) of all the cases had protein plugs, among which, 38.0% (8/21) presented with recurrent acute pancreatitis. For pediatric cases, 36.9% (17/46) had protein plugs, 29.4% (5/17) presented with recurrent acute pancreatitis.

Endoscopic treatments for PBM primarily include EST, stent insertion and ENBD/ENPD. If pancreaticobiliary stones or protein plugs were detected, stone extraction would be needed. Published literature on the use of ERCP in PBM is limited to small series. Ng et al. [2] first reported 6 PBM patients who underwent EST, five got satisfactory outcomes. Similarly, Guelrud et al. [3] showed that following a mean 26.4 months in 9 PBM patients who underwent ERCP therapy, eight had excellent results. Subsequently, Samavedy et al. [4] reported 15 PBM patients over 10-year period, thirteen of them benefited from ERCP therapy during 3 years follow-up. Recently, a relatively large study of 19 pediatric patients with PBM has suggested ERCP can be a logical first step in the management of most symptomatic patients with PBM [8]. In contrast to previous studies, our study is the largest study describing the utility of ERCP in PBM to the best of our knowledge. Apart from adding evidence to the reported advantages of ERCP, we attempt to answer some of the uncertainties about it.

Our study showed that ERCP was an effective treatment option for PBM patients accompanied by biliary obstruction. Patients benefitted from ERCP with significant decline in the levels of elevated liver enzymes and bilirubin. However, a trend towards increase in the level of serum amylase was observed. It could be related to asymptomatic hyperamylasemia, a common condition ranging from 16.5% to 18.3% after ERCP [20,21]. Our study also showed that patients who experienced recurrent pain attacks achieved impressive relief after ERCP therapy. Significant decline was identified in severity and frequency of abdominal pain during follow-up. The total effective rate of ERCP therapy was 60.7%, which meaned 34 of 56 patients reported improvement of general condition after their index ERCP, without required readmission or further endoscopic intervention. We did not observe significant change in frequency of recurrent pancreatitis, this could be due to the short follow-up duration and low frequency of pancreatitis attacks. Therefore, long-term follow-up studies are needed to verify any true effects of ERCP therapy on pancreatitis attacks of PBM patients.

It is reported that the overall incidence of biliary carcinoma with PBM is more than 200 times higher comparing to the risk in the general population [17]. The age at which PBM patients become predisposed to develop biliary carcinoma is around 50-65 years [1]. During follow-up, our cohort had two elderly female patients without congenital biliary dilatation suffered from associated gallbladder cancer. Established management of PBM without congenital biliary dilatation is prophylactic cholecystectomy. Based on our experience, a sphincterotomy before cholecystectomy may prevent pancreaticobiliary reflux and help reduce the risk of malignancy for PBM patients without congenital biliary dilatation. Another research trying to verify this hypothesis is in progress.

Preoperative ERCP may benefit PBM patients at the following 4 points. (1) ERCP provides detailed information on the pancreaticobiliary systems, rules out other possible pancreaticobiliary anomalies, and helps to decide on the appropriate surgical strategy. (2) A better physical status is achieved preoperatively. Biliary obstruction can be treated by ERCP. (3) Recurrent pain and pancreatitis attacks among PBM patients may be attributed to sphincter of Oddi dysfunction and protein plugs incarceration, ERCP is an ideal method to resolve these problems. (4) For PBM patients without congenital biliary dilatation, a sphincterotomy before cholecystectomy may help reduce the risk of malignancy.

According to the ASGE ERCP difficulty grading system, 37.8% (28/74) of our ERCP procedures were evaluated as 3^rd^ level and 4^th^ level. This was because our series contained a large proportion of children and patients who need interventions in pancreatic diseases. The difficulty increases, the technical success rate decreases and the complication rate increases. Our technical success rate was 97.3%. Two cases were failed because of complicated pancreaticobiliary ductal union. The overall frequency of PEP was 4.1%, higher than the 2.6% reported in unselected series [22]. This might be related to the higher portion of patients with pancreatitis history in our study, in whom PEP is more common. Hemorrhage occurred in 2.7% of all cases, was mild and managed conservatively. Considering the difficulty and risk, the ERCP treatment for PBM should be performed by experienced endoscopists in advanced center.

Ng [2] and Terui [7] have speculated that EST was less effective in PBM patients with undilated common channel, however, the sample size was quite small. Thus we performed a further analysis to compare the benefit of EST on patients with undilated common channel and patients with dilated common channel. The results showed that the baseline characteristics, effective rate, general condition, adverse events and follow-up results had no difference between the two groups. It can be concluded that efficacy and safety of EST in PBM patients with undilated common channel was comparable to that in patients with dilated common channel.

Our study had several limitations. Firstly, the retrospective and single-centred design increased the likelihood for recall bias and selection bias. Secondly, parameters in our study such as general condition and pain situation were subjective. Thirdly, the small number of patients who underwent EST may limit the reliability of statistical analysis.

In conclusion, ERCP can serve as a transitional step to definitive surgery for PBM patients. It can guarantee pancreaticobiliary drainage and relieve clinical symptoms, only with a low incidence for mild complications. PBM patients with undilated common channel could benefit from EST as well as patients with dilated common channel. Further studies with greater sample sizes were warranted.
